# The Movember Prostate Cancer Landscape Analysis: an assessment of unmet research needs

**DOI:** 10.1038/s41585-020-0349-1

**Published:** 2020-07-22

**Authors:** Michelle M. Kouspou, Jenna E. Fong, Nadine Brew, Sarah T. F. Hsiao, Seanna L. Davidson, Peter L. Choyke, Tony Crispino, Suneil Jain, Guido W. Jenster, Beatrice S. Knudsen, Jeremy L. Millar, Nicole Mittmann, Charles J. Ryan, Bertrand Tombal, Mark Buzza

**Affiliations:** 1 Movember, Melbourne, Victoria Australia; 2 The Systems School, Melbourne, Victoria Australia; 3 Center for Cancer Research, National Institute of Health/National Cancer Institute, Bethesda, MD USA; 4 SWOG Cancer Research Network, Prostate Cancer Patient Advocate, Portland, OR USA; 5 Patrick G Johnston Centre for Cancer Research, Queen’s University Belfast, Belfast, UK; 6 Department of Urology, Erasmus Medical Center, Rotterdam, Netherlands; 7 University of Utah, Salt Lake City, UT USA; 8 Alfred Health Radiation Oncology, The Alfred Hospital School of Science, Melbourne, Victoria Australia; 9 RMIT University, Melbourne, Victoria Australia; 10 Sunnybrook Research Institute, Sunnybrook Health Science Centre, University of Toronto, Toronto, Ontario Canada; 11 University of Minnesota, Minneapolis, MN USA; 12 Université Catholique de Louvain, Louvain-la-Neuve, Belgium; 13 European Organisation for Research and Treatment of Cancer, Brussels, Belgium

**Keywords:** Prostate cancer, Prostate cancer, Health policy, Public health

## Abstract

Prostate cancer is a heterogeneous cancer with widely varying levels of morbidity and mortality. Approaches to prostate cancer screening, diagnosis, surveillance, treatment and management differ around the world. To identify the highest priority research needs across the prostate cancer biomedical research domain, Movember conducted a landscape analysis with the aim of maximizing the effect of future research investment through global collaborative efforts and partnerships. A global Landscape Analysis Committee (LAC) was established to act as an independent group of experts across urology, medical oncology, radiation oncology, radiology, pathology, translational research, health economics and patient advocacy. Men with prostate cancer and thought leaders from a variety of disciplines provided a range of key insights through a range of interviews. Insights were prioritized against predetermined criteria to understand the areas of greatest unmet need. From these efforts, 17 research needs in prostate cancer were agreed on and prioritized, and 3 received the maximum prioritization score by the LAC: first, to establish more sensitive and specific tests to improve disease screening and diagnosis; second, to develop indicators to better stratify low-risk prostate cancer for determining which men should go on active surveillance; and third, to integrate companion diagnostics into randomized clinical trials to enable prediction of treatment response. On the basis of the findings from the landscape analysis, Movember will now have an increased focus on addressing the specific research needs that have been identified, with particular investment in research efforts that reduce disease progression and lead to improved therapies for advanced prostate cancer.

## Abstract (web summary)

The Movember global Landscape Analysis Committee (LAC) was established to act as an independent group of experts across urology, medical oncology, radiation oncology, radiology, pathology, translational research, health economics and patient advocacy to identify the highest priority research needs across the prostate cancer biomedical research domain. Findings from the landscape analysis illustrate the research priorities in prostate cancer and will enable Movember to focus on specific needs, with particular investment in research to reduce disease progression and improve therapies for advanced prostate cancer.

## Introduction

Prostate cancer is the second most diagnosed solid-organ malignancy in men^1^, with >1.2 million new cases and 358,989 deaths reported worldwide in 2018 (ref.^2^). Since 2005, the global men’s health charity Movember has invested in a variety of very influential programmes in biomedical research, survivorship and clinical quality and has set ambitious organizational objectives to work with its global partners to halve the number of deaths from prostate cancer and to halve the number of men facing ongoing adverse effects from treatment by 2030. In order to transform these aspirations into reality, Movember needs to understand the gaps and opportunities in the field.

Given that the last published analysis of the field was conducted by Tindall and Scardino^3^ ~20 years ago, Movember’s Global Scientific Committee (GSC) recommended that the Movember Biomedical Research Team commission a formal landscape analysis to assess the international prostate cancer research field in order to optimize future research investment and maximize biomedical research outcomes. The scope of this analysis was restricted to the role of Movember as a funder whose purpose is to positively change clinical practice in the prostate cancer biomedical research field for the benefit of men.

In this Consensus Statement, we present the results and conclusions of Movember’s prostate cancer global landscape analysis.

## Methods

**Fig. 1:**
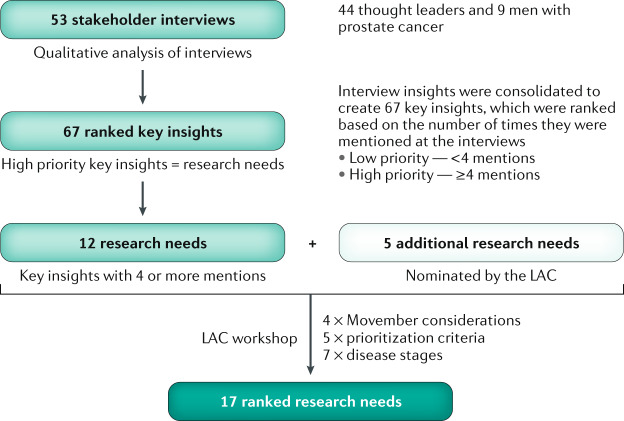
Overview of the landscape analysis process. The landscape analysis methodology involved one-to-one interviews, qualitative analysis and consultation with international experts (the Landscape Analysis Committee (LAC)). The research needs were then ranked using agreed prioritization criteria at the LAC workshop meeting.

The Movember prostate cancer landscape analysis commenced in April 2017. A sequential research design was used, in which insights gathered from stakeholder interviews were collated, prioritized and discussed during a facilitator-led expert workshop for consensus in December 2017 (Fig. 1). First, men with prostate cancer were invited to provide their personal experience and reflections via in-depth interviews with members of the Movember Biomedical Research Team (M.B. and M.M.K.). A diverse range of thought leaders were also interviewed to gauge their views on the current state of prostate cancer research, funding climate and the optimal role for Movember in the global research landscape. Interview responses were collated and analysed to create a list of insights ranked by response frequency. An expert Landscape Analysis Committee (LAC) was convened in order to evaluate the highest-ranked insights against research prioritization criteria, which were based on LAC consensus and were defined and explained to the LAC workshop participants in advance. In total, five thematic areas were considered for prioritization: health outcomes, research significance, implementation, equity and the state of current research (Table 1). Insights were subsequently ranked as research needs. Process design and workshop facilitation was supported by an independent systems research consultant (S.L.D.).

**Table 1:** Research prioritization criteria

Thematic area	Criteria	Definition	Refs
Health outcomes	Improved patient QOL	The research has the potential to improve the QOL of a patient within a specific step of the treatment journey and/or improved QOL during and beyond the treatment process	^8^
Research significance	Reduces burden of disease	The research has the potential to markedly reduce the burden of disease. Research needs to be important, innovative, non-redundant and address a key research gap	^9,12^
Implementation	Implementable	The barriers to implementation are not insurmountable (the research is technically and practically feasible), and the research has the potential to move to the next stage of development (is translational or clinical)	^8^
Equity, ethics, fairness	Health inequities	The research has the potential to positively impact and be accessible to a diverse range of men across a diverse range of geographies	^10,11^
State of current research	Significant current momentum	Research in the field that could enable significant improvement in the research need, if Movember were to support it	NA

NA, not applicable; QOL, quality of life.

### Benchmarking other landscape analyses

In total, three highly relevant landscape analyses were benchmarked — Tindall and Scardino’s original prostate cancer landscape analysis^3^, the Metastatic Breast Cancer Landscape Analysis research report from the Metastatic Breast Cancer Alliance^4^ and the Cancer Moonshot Blue Ribbon Report^5^ — to understand the methodological approaches that were used, garner key lessons learned and adopt best practice. The approaches were reviewed and adapted to reflect Movember’s role as a backbone organization as part of a collective impact model in the broader prostate cancer biomedical research setting^6,7^.

### Stakeholder interviews

M.M.K. and M.B. conducted a total of 53 in-depth one-on-one interviews with men with prostate cancer (*n* = 9) and global thought leaders in the field of prostate cancer research (*n* = 44) to gather a broad range of insights to be used as context and content for the LAC workshop (Box 1). Using a modified human-centred approach, nine men from Australia, the USA and Europe with disease ranging from localized with no progression after primary treatment to advanced metastatic, shared their personal experiences of living with prostate cancer, including diagnosis, treatment and support received in both clinical and survivorship care, awareness of clinical trials available and the most considerable challenges and/or barriers. The interviews with the men with prostate cancer also included standardized questions and queries about gaps or roadblocks that prevent advances in the prostate cancer research space and how they could be addressed from a patient’s perspective, aspects of biomedical prostate cancer research that Movember should invest in that would have helped with their own experiences with prostate cancer, and what success would look like if the endeavour were to be successful. Additionally, 44 thought leaders from a broad range of relevant disciplines (including health economists, epidemiologists, urologists, medical oncologists, radiation oncologists, pathologists, foundation and health organization representatives, research academics and industry representatives) were invited to provide their professional opinion in response to a list of standardized questions, which included queries about the future of prostate cancer research and health care over the next 3–5 years; where there is existing momentum; the research gaps that need addressing to move the field forwards; where Movember should invest over the next 3–5 years to have the greatest influence; how Movember can best coordinate a collective approach to consolidate the field and maximize patient outcomes; where other funders are investing in the space and the current barriers to conducting clinical trials in prostate cancer (Box 2).

Standardized questions were developed by benchmarking and reviewing earlier landscape analyses^3–5^ and direct discussions with the programme leaders who conducted previous prostate cancer and Metastatic Breast Cancer Landscape analyses^4^. The questions were reviewed and endorsed by the Movember GSC, which comprises global thought leaders in prostate cancer research.

At the commencement of all interviews, an outline of Movember’s role as a not-for-profit organization with limited resources to maximize patient outcomes was provided for context.

Upon completion of the interviews, all responses were analysed qualitatively and consolidated using a thematic approach to generate a list of key insights ranked by the response frequency (the number of times the insight was mentioned by the interviewees). For the purposes of generating a shortlist of research needs, only those key insights mentioned by four or more interviewees were prioritized for discussion at the LAC workshop (*n* = 12; referred to as ‘research needs’ hereafter). The members of the LAC were asked to draw on their own expertise and collectively determined that the shortlisted 12 research needs were not entirely sufficient to describe all of the critical unmet research needs in the field. Thus, the LAC reviewed all 67 insights gathered from the interviews and, based on a consensus approach, determined that one lower-ranked insight (in terms of the number of times mentioned) should also be included for prioritization review. Four additional research needs were nominated and agreed by the LAC. All 17 (12 + 5) were reviewed and agreed upon amongst all LAC members before the face-to-face workshop.

Box 1 List of stakeholders involved in the landscape analysis **Interview stakeholders**

- Men with prostate cancer (9 men)
- Health economists (3 individuals)
- Epidemiologists (3 individuals)
- Urologists (8 individuals)
- Medical oncologists (6 individuals)
- Radiation oncologists (3 individuals)
- Pathologists (2 individuals)
- Foundation or health organization representatives (7 individuals)
- Research academics (9 individuals)
- Industry representatives (3 individuals)

**Landscape Analysis Committee members**

- Peter L. Choyke (USA), Chief, Molecular Imaging Program, Center for Cancer Research, National Cancer institute, National Institutes of Health
- Suneil Jain (UK), Clinical Reader and Honorary Consultant in Clinical Oncology, Patrick G Johnston Centre for Cancer Research, Queen’s University Belfast
- Guido W. Jenster (Netherlands), Professor, Experimental Urological Oncology; Director, Experimental Urology Laboratory, Erasmus MC
- Bertrand Tombal (Belgium), Chairman and Professor, Division of Urology, Université Catholique de Louvain, Cliniques Universitaires Saint-Luc in Brussels; President, European Organization for Research and Treatment of Cancer
- Beatrice S. Knudsen (USA), Professor, Pathology, University of Utah; Medical Director of Digital and Computational Pathology, ARUP national reference laboratory
- Charles J. Ryan (USA), Professor, Medicine, Division of Hematology, Oncology and Transplantation, University of Minnesota
- Jeremy L. Millar (Australia), Director, Radiation Oncology Alfred Health; Professor, Central Clinical School, Monash University; Clinical Lead, Prostate Cancer Outcomes Registry, Monash University; Deputy Chair, Cancer Council Australia; Director, Cancer Council Australia
- Tony Crispino (USA), Prostate Cancer Survivor; President — Us TOO Prostate Cancer Support and Education Las Vegas Chapter; SWOG Cancer Research Network Patient Advocate
- Nicole Mittmann (Canada), Chief Scientist and Vice-President of Evidence Standard, Canadian Agency for Drugs and Technologies in Health and Department of Pharmacology and Toxicology, University of Toronto

Names listed in the Acknowledgements section.

Box 2 Interview questions and LAC workshop discussion topics **Interview questions for men with prostate cancer**

Invite men to share their experience with prostate cancer, including diagnosis, treatment and support received in both clinical and survivorship care, awareness of clinical trials available and most significant challenges and/or barriers. Ask each man a series of standardized questions:

1. What are the gaps or roadblocks that prevent advances in the prostate cancer space and what do men think could be done to address the gaps?
2. What aspects of biomedical prostate cancer research should Movember invest in that would have helped their experience with prostate cancer?
3. What would success look like in 5 years’ time?

**Thought leader interview questions**

1. Where is the field of prostate cancer research and health care likely to be over the next 3–5 years?
2. Where is the existing momentum?
3. What are the research gaps that need addressing to move the field forwards?
4. Where should Movember invest over the next 3–5 years to have the greatest impact?
5. Where can Movember best coordinate a collective impact-style approach to consolidate the field and maximize patient outcomes?
6. In what areas are other funders investing in the space?
7. What are current barriers in conducting clinical trials in prostate cancer?

**Movember considerations and discussion at workshop**

1. What would success look like in 5 years’ time?
2. What are the existing barriers in the research field?
3. What would be the optimal investment strategy?
4. How could Movember collaborate with other funders to exert a collective impact in the field?

LAC, Landscape Analysis Committee.

### Landscape Analysis Committee

Towards the end of the interview process, a LAC comprising global thought leaders was assembled by Movember. The aim was for the committee to meet face-to-face as an expert group to review the key insights and research needs identified from the stakeholder interviews in the context of agreed research prioritization criteria. Representation of the following disciplines was deemed crucial: urology, medical oncology, radiation oncology, radiology, pathology, academic translational research, health economics and research implementation, and patient advocacy. Members of the LAC were considered to be thought leaders in their field and a balance was sought between discipline, geographical region and gender. A focus of the landscape analysis was prostate cancer biomedical research, but several LAC members also had strong expertise in prostate cancer clinical quality and/or patient survivorship.

The composition of the final LAC was Bertrand Tombal (urology, Belgium), Charles J. Ryan (medical oncology, USA), Beatrice S. Knudsen (pathology, USA), Suneil Jain (clinical oncology, UK), Jeremy L. Millar (radiation oncology, Australia), Peter L. Choyke (radiology, USA), Guido W. Jenster (translational research, Netherlands), Nicole Mittmann (health economics and policy, Canada), Tony Crispino (patient advocacy, USA) (Box 1).

A face-to-face workshop facilitated by a systems research consultant (S.L.D) was held in Los Angeles in December 2017, with the aim of interrogating the identified research needs in the context of a set of agreed research prioritization criteria (Table 1) and rank the research needs for future consideration of Movember funding. These prioritization criteria included the ability to improve patient quality of life (QOL), reduce the burden of disease, be implementable, address health inequities, and have significant current momentum, and were agreed via a consensus process before the face-to-face workshop. Each prioritization criterion (which considers the broad context outside of biomedical research) was applied equally to all identified research needs through multiple rounds of group discussion at the workshop.

### Establishing prioritization criteria

As part of the quality assurance process and to establish appropriate research prioritization criteria that would enable identification of the most pressing research needs requiring potential future research investment, a review of the literature on research prioritization criteria and processes was conducted in order to benchmark best practice. The review was conducted in Scopus in mid-2017 and terms included ‘criteria’ AND ‘health’ AND ‘priority/priorities’ AND ‘investment’. From the 125 publications returned using the search terms, a review of the abstracts for appropriateness identified 16 publications of relevance. Five publications were ultimately deemed to be the most relevant to the needs of men with prostate cancer^8–12^.

Two approaches were identified regarding how to develop the prioritization criteria. In a systematic review of multi-criteria decision analyses, Cromwell et al.^13^ identified a set of common domains that were used to group decision-making criteria. A review of the CHNRI method for setting health research priorities suggested prioritization based on the 4D method (description, delivery, development, discovery), whereby potential research avenues are first categorized by type of research instrument and the decision-making criteria are applied directly to the proposed research avenues^11^. The method outlined by Cromwell et al. was selected and applied to identify a set of themes to be evaluated, with each theme including specific criteria. In brief, M.B., M.M.K. and S.L.D. reviewed the most common domains and criteria and clarified their definition from the literature, which was used to ascertain a shared understanding for the application process. The initial criteria list was further refined as suggested by Marsh et al.^14^, whereby considerations were given to the properties of: completeness — criteria should capture all factors relevant to the decision; non-redundancy — criteria should be removed if they are unnecessary or judged to be unimportant; non-overlap — criteria should be defined to avoid double counting; and preference independence — how much one cares about the performance on a criterion should not depend on the performance of other criteria. Based on the logic derived from available research best practice, the list of prioritization criteria was finalized with the LAC via teleconference, ahead of the face-to-face workshop (Table 1).

### Face-to-face workshop

The workshop began with four rounds of group dialogue that helped to define the context of this landscape analysis initiative and encouraged the participants to consider Movember’s perspective as a research funder to maximize outcomes and patient benefit from its prostate cancer biomedical research investments. The considerations for Movember were: what would success look like in 5 years’ time? What are the existing barriers in the research field? What would be the optimal investment strategy? And how could Movember collaborate with other funders to exert a collective impact on the field? (Box 2).

LAC members then independently evaluated all research needs against the five agreed prioritization criteria to determine their importance, marking each with a “yes” (important), “no” (not important) or “not qualified to answer”^11^. In a facilitated group dialogue, any research need that had a low level of agreement was re-evaluated and the rationale that supported and/or opposed each criterion was discussed. A second round of scoring was then conducted and a new count was carried forwards for final consideration. Thus, each research need received a prioritization score between 0 and 5, in which a score of 1 was allocated to each “yes” and a 0 score allocated for each “no” or “not qualified to answer” (Table 2; Fig. 1).

**Table 2:** Research needs by disease stages

Disease stage	Research need number	Research need description	Interview mentions	LAC prioritization score
Screening and early diagnosis		Establish more sensitive and specific tests to improve disease screening and diagnosis	6	5
Localized disease		Develop indicators to better stratify low-risk prostate cancer in determining which men should go on active surveillance	18	5
		Standardized active surveillance guidelines to aid decision-making	4	4
		Educate men about the benefits of active surveillance and ways of reducing anxiety	4	4
		Improve current standard-of-care treatment (radiotherapy and surgery) to maximize patient mental and physical well-being	NA	3
		Determine how the interplay between genetics and lifestyle impacts disease progression	<4	1
Locally advanced disease		Determine the most effective way of treating biochemical recurrence in order to improve survival and enhance quality of life	NA	3
		Improve the use of androgen deprivation therapy to minimize adverse effects of treatment	4	3
		Perform genomic profiling early to optimize treatment and identify likely responders	5	3
Oligometastatic disease		More accurately define oligometastatic prostate cancer and determine the best treatment strategy	7	4
Advanced disease		Determine the optimal treatment sequence for men with mCRPC that will lead to the best outcomes for a man’s specific tumour type	4	3
		Determine the potential of immunotherapy as a treatment option for mCRPC	6	1
Disease biology		Better understand the biology of disease progression in order to optimize treatment decisions	5	3
Treatment optimization		Further progress precision medicine such as use of biomarkers that can enable personalized treatment decision	32	3
		Integrate companion diagnostics (for example, liquid and/or tissue biopsy and imaging modalities) into randomized clinical trials to predict treatment response	5	5
		Demonstrate clinical utility of validated liquid biopsies	NA	4
		Replace tissue biopsies with non-invasive biomarkers (such as liquid biopsy or imaging modalities)	NA	3

Research needs in a solid circle are insights from the stakeholder interviews that were prioritized for workshop discussion (those mentioned by four or more interviewees); research needs in an open circle are the LAC's recommendation for discussion. LAC, Landscape Analysis Committee; mCRPC, metastatic castration-resistant prostate cancer; NA, not available.

## Analysis and discussion

**Fig. 2:**
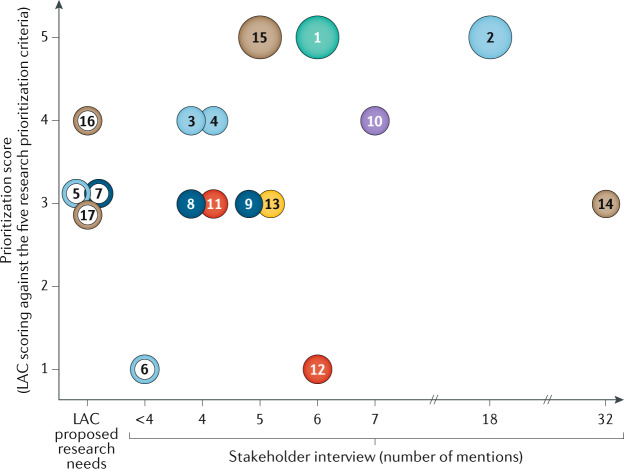
Summary of prioritized research needs from the landscape analysis. Each research need, ordered according to its relevant disease stage, was plotted by the number of times it was mentioned in the thought leader and patient interviews (*x* axis) against the prioritization score it was given by the Landscape Analysis Committee (LAC; *y* axis). Research needs in a solid circle are insights from the stakeholder interviews that were prioritized for workshop discussion (those mentioned by four or more interviewees); research needs in an open circle are the LAC’s recommendation for discussion.

The stakeholder interview rankings were plotted against the prioritization scores to identify the most important research needs (Fig. 2). From this analysis, 17 research needs were identified by the LAC. The highest scoring research needs were in the treatment optimization, screening and early diagnosis, localized disease and oligometastatic disease fields (each of these needs were mentioned at least four times during stakeholder interviews and received a prioritization score of 4 or 5 by the LAC). These fields are predominantly (but not solely) applicable to the early stages of prostate cancer, which affects the most men and their families.

Of the identified research needs, the maximum prioritization score of 5 was received by research needs number 1: establish more sensitive and specific tests to improve disease screening and diagnosis; number 2: develop indicators to better stratify low-risk prostate cancer in determining which men should go on active surveillance (AS), and number 15: integrate companion diagnostics into randomized clinical trials to enable prediction of treatment response. These highest priority research needs are discussed in detail to highlight the identified gaps and outline potential opportunities for future collective effort.

Research need number 14 (further progress precision medicine) was mentioned considerably more often than others during the stakeholder interviews (32 mentions) and certainly reflects the current view of the research community, with approximately 500 review articles on “prostate cancer precision medicine” published between 2017 and 2019. However, whilst the notion of offering a man with prostate cancer the most effective medicine at the optimal time based on his individual genomic profile is highly desirable, precision medicine only received an overall prioritization score of three as a research need, as the LAC did not feel it could be strongly supported by the current state of knowledge (research prioritization criteria: implementable) nor would it particularly help to address the research prioritization criteria around health inequities. This thinking continues to be relevant, as despite progress in the field of precision medicine in prostate cancer (such as the FDA approval of PARP inhibitors for men with germline or somatic homologous recombination repair (HRR) gene mutations^15,16^), considerable effort is still needed for precision oncology to be broadly applicable to men around the world and for validated biomarkers to be capable of realizing a truly personalized treatment approach^17^. It should be noted that, although the concept of ‘health inequities’ was an agreed prioritization criterion and was a component of the LAC prioritization discussions at the face-to-face workshop, it was not a component of the stakeholder interviews per se. During the workshop, research need number 14, was deemed incredibly important, but was considered to have a low capacity to address health inequities on a global scale, particularly owing to the high pricing of precision tests and biomarkers and accompanying novel therapies in the USA. Without sufficient nationwide health insurance in the USA, precision medicine elements such as biomarkers, novel imaging agents and some novel therapies were thought to be very often unaffordable for many patients. Patient access to and reimbursement of new oncology therapies is varied in Organisation for Economic Co-operation and Development countries and can take many years after first marketing approval is granted, typically in the USA^18^.

Opportunities to prevent disease progression to life-threatening advanced disease exist during the transitional state between localized disease and oligometastatic disease. Research need number 10 (to more accurately define oligometastatic prostate cancer and determine the best treatment strategy) was highly prioritized by the LAC (Fig. 2) and is a very active research topic with numerous important clinical trials being conducted in this area (such as SWOG S1802 (ref.^19^), ORIOLE^20^, STOMP^21^, RAVENS^22^, STORM^23^, FORCE^24^). It is hoped that these trials will lead to enhanced detection of disease recurrence, as well as improved use of biological and/or imaging biomarkers, enabling the delivery of potentially curative treatments, which will reduce the number of men progressing to more advanced and lethal disease.

## Perspectives of men with prostate cancer

Interviews revealed that living with prostate cancer can considerably affect a man’s physical and mental well-being. In general, men were satisfied with the quality of the health-care services they received. Connecting with other men with prostate cancer was generally thought to be highly valuable by the men who were interviewed; however, the selected interviewees’ views might not be reflective of all men with prostate cancer. Feedback from interviewees suggested that the provision of quality health information to support improved informed decision-making was crucial for men to feel an increased sense of ownership of their own health. Additionally, Movember was thought to be best placed to further support this provision by raising awareness of prostate cancer and ensuring that high-quality information is available for the general public. Men highlighted that there were elements of confusion during particular stages of disease management. Specifically, they mentioned the need to improve clarity of population screening guidelines, the need for improved diagnostic tools to distinguish aggressive and indolent prostate cancer, the need to standardize treatment approaches, and necessary improvements in the broader implementation of treatment guidelines within their own countries. One man also suggested that clinical trials in prostate cancer could be conducted more efficiently by improving clinical trial networks. Other suggestions to improve research included exploring the repurposing of existing drugs and research that will minimize the long-term use of androgen deprivation therapy (ADT) and its associated adverse effects. Men also suggested that measures to reduce racial, socioeconomic and regional disparities should be embedded into the delivery of research and health care. Lastly, it was suggested that Movember should continue to engage with other funders, governments and industry to promote synergies, maximize research impact and avoid duplication of effort.

## Consensus on identified research needs

The culmination of the landscape analysis was consensus on 17 research needs in the field of prostate cancer research. The LAC scored each research need against the five prioritization criteria (Fig. 2), which have been reviewed in detail by Movember. Future investments by Movember into new prostate cancer biomedical research initiatives that address these different research needs will be made in the context of existing programmatic investments, as well as ongoing and planned programmatic investments by other global funding bodies, with the aim of reducing duplication of effort and maximizing patient outcomes. The 17 research needs are described and categorized based on their relevant stage of disease.

### Screening and early diagnosis

Early identification of cancer typically enables improved implementation of treatment options, resulting in improved clinical outcomes, including increased overall survival. However, without a reliable, minimally invasive test for risk assessment and diagnosis of clinically significant prostate cancer, population-based screening will likely subject too many men to unnecessary treatment and avoidable adverse effects, with the risk of aggressive disease still being overlooked. Thus, the need to establish more sensitive and specific tests to improve disease screening and diagnosis (research need 1; LAC score 5) was highly prioritized by the LAC. Inclusion of diagnostic parameters in prostate cancer risk calculators, improved use of novel imaging to improve biopsy accuracy, examination of germline mutations to identify high-risk subpopulations, or adoption of a suite of molecular urine and blood biomarkers are all opportunities that should be explored.

### Active surveillance

Safely delaying or avoiding unnecessary treatment for a select population of men with low-risk (and potentially intermediate-risk) prostate cancer through AS is widely accepted as a suitable management approach. However, uncertainty still remains regarding which patients should be enrolled to AS protocols, the optimal follow-up schedule for patients based on individual risk, the best strategies to mitigate anxiety and reduce the number of men switching to active treatment if they are not progressing, as well as the economic cost benefits and value. In order to improve management of men with clinically insignificant disease on AS, it was deemed critical to develop indicators to better stratify low-risk prostate cancer and help determine which men should go on AS (research need 2; LAC score 5), standardize AS guidelines to aid decision-making (research need 3; LAC score 4) and educate men about the benefits of AS and ways of reducing anxiety (research need 4; LAC score 4).

### Localized disease

Men diagnosed with high-risk prostate cancer are generally offered surgery and/or radiotherapy with a curative intent in the Organisation for Economic Co-operation and Development countries^25^. However, the survival benefits from these primary treatments are also associated with significant adverse side effects, including urinary, sexual and bowel dysfunction^25^. Improving current standard-of-care treatment to maximize men’s mental and physical well-being (research need 5; LAC score 3) was identified as an unmet research need to help mitigate the substantial patient burden of these common adverse outcomes. During the interview process, some stakeholders suggested that determining the interplay between genetic and lifestyle factors in driving disease progression (research need 6; LAC score 1) should be a key research focus, but this was not ranked a high priority by the LAC.

### Locally advanced disease

Approximately 20–40% of men who undergo either surgery or radiation therapy progress to a state of biochemical recurrence, as signified by a rise in their serum PSA level^26,27^. Advancement in novel imaging technologies, including multiparametric MRI (mpMRI) and novel PET tracers have revolutionized the diagnosis of biochemical recurrence^28,29^. Lesions located in the prostate bed or nodal, bony or visceral sites can now potentially be detected in men using novel imaging long before their PSA level reaches the threshold of 0.2 ng/ml (ref.^30^). However, the ability to detect disease recurrence much earlier using imaging now means that conventional treatments that would have previously been prescribed without this additional information, might no longer be adequate or optimally timed. Thus, the LAC agreed that it is important for the field to use these new tools to determine the most effective way of treating biochemical recurrence in order to improve survival and enhance QOL (research need 7; LAC score 3) but also to improve the use of ADT to minimize treatment-related adverse effects (research need 8; LAC score 3). The LAC also felt that in the locally advanced setting, there was an opportunity to perform genomic profiling earlier in the therapeutic pathway in order to optimize treatment and identify likely responders to therapy (research need 9; LAC score 3).

### Oligometastatic disease

Advances in novel imaging techniques have also enabled accelerated research progress and knowledge in the field of oligometastatic prostate cancer. Consensus on defining the oligometastatic disease state and the opportunity it presents to potentially ‘cure’ the disease remain contentious within the research and clinical community^31^. Aggressive metastasis-targeted approaches are being extensively investigated to understand their efficacy to ablate detected lesions and reduce the spread of further metastases^32,33^. Some experts believe that oligometastatic disease is simply an indicator that the disease has progressed to be systemic and is no longer amenable to metastasis-targeted therapy^31^. Targeted, high-dose radiation with stereotactic ablative radiotherapy is emerging to be a safe and effective option in delaying systemic ADT with or without chemotherapy^34^. Further large-scale studies that validate the findings from early stereotactic ablative radiotherapy trials and enable a more accurate definition of oligometastatic prostate cancer and determine the best treatment strategy (research need 10; LAC score 4) was very highly prioritized by the LAC.

### Advanced disease

Men with advanced prostate cancer face a poor prognosis and increased risk of treatment-incurred adverse effects. Targeting the androgen receptor (AR) signalling pathway remains the most universally effective means of achieving disease control. Results from large phase III trials have demonstrated that men with hormone-sensitive prostate cancer treated with novel AR-targeted therapies and ADT^35–37^ do better than men treated with ADT alone, which has now become standard of care. However, only a limited number of treatments are available once men progress on ADT and, depending on which therapy has been used in the hormone-sensitive stage, available treatment choices for men with metastatic castration-resistant prostate cancer (mCRPC) can still be limited^38,39^. Studies are already underway to address this critical challenge, including a crossover trial that has provided essential evidence that the sequence of abiraterone (plus prednisone) before enzalutamide was associated with longer time to second PSA progression than the reverse sequence^40^. Comprehensive studies will be required in order to determine the optimal treatment sequence for men with advanced disease that will lead to the best outcomes for a man’s specific tumour type (research need 11; LAC score 3). Clinical quality registries, such as the Movember-funded IRONMAN study, will also help to address this question and ultimately lead to better outcomes for men with advanced prostate cancer^41^.

A variety of novel approaches continue to be explored in the advanced disease setting. In addition to promising outcomes with PARP inhibitors^42,43^ and PSMA-targeted theranostics^44^, the potential of immunotherapy as a treatment option for men with mCRPC (research need 12; LAC score 1) is of particular interest, despite discouraging initial trial results in prostate cancer compared with the effects in other tumour types. Efficacy and safety of various immunotherapies, including vaccines, immune checkpoint inhibitors, virus-mediated immune modulation and adaptive T cell therapy, are being investigated as monotherapies or combination therapies. As of April 2020, a search for “prostate cancer metastatic” AND “immunotherapy” revealed 34 active clinical trials on clinicaltrials.gov (including trials that are ‘recruiting’, ‘not yet recruiting’ and ‘active not recruiting’). Although these trials are still years away from revealing any meaningful clinical outcomes, substantial progress can be expected in this field over the next decade to unravel ways of priming the tumour immune response for effective immunotherapies^45^. The LAC felt that the development of such agents should be the realm of industry rather than funding bodies such as Movember.

### Disease biology and treatment optimization

Considerable improvement in prostate cancer treatment and management has been made over the past decade following discoveries in biomedical and clinical research. These discoveries have been realized through continuous investigation of key aspects of the underlying biology of the disease. As health care enters the era of precision medicine, an improved understanding of the biology of disease progression will be important for optimizing treatment decisions (research need 13; LAC score 3). Owing to the critical role of biomarkers in enabling precision medicine, their development and clinical utility constituted a strong theme running through this landscape analysis. Thus, the need to further progress precision medicine, such as the use of biomarkers that can enable personalized treatment decisions (research need 14; LAC score 3), was identified as a key area of research warranting further investigation and funding, during stakeholder interviews and by the LAC. Furthermore, integration of companion diagnostics (for example, liquid and/or tissue biopsies and imaging modalities) into randomized clinical trials to predict treatment response (research need 15; LAC score 5) scored the maximum prioritization score by the LAC. Although a large number of liquid biopsies have been developed (or are currently in development) to better inform patients and clinicians about a tumour’s clinical significance and current state of progression, few have effectively entered the mainstream health-care system to a point where they are being routinely used to optimize treatment decisions as a universal standard of care^46^. A critical next step is, therefore, to demonstrate the utility of these validated liquid biopsies (research need 16; LAC score 4) if they are to have a real effect on the health outcomes of all men, irrespective of their region or socioeconomic status. One specific area that the LAC felt had considerable potential for a positive effect was to replace tissue biopsies with non-invasive biomarkers, such as liquid biopsy or imaging modalities (research need 17; LAC score 3), but, as outlined above, this endeavour is not without significant challenges.

## Highest priority research needs

Maximum prioritization (a score of 5) was given to three research needs by the LAC. The highest priority research needs are research need number 1: establishing more sensitive and specific tests to improve low-risk disease screening and diagnosis; research need number 2: developing indicators to better stratify low-risk prostate cancer in determining which men should go on AS; and research need number 15: integration of companion diagnostics in randomized clinical trials to enable prediction of treatment response. These research needs have been considered in a contemporary context.

### Research need number 1

#### Establishing more sensitive and specific tests to improve low-risk disease screening and diagnosis

PSA screening is a highly controversial topic in urology; screening guidelines are often not population-based and vary in their recommendations in different jurisdictions^47^. Virtual consensus is exhibited across all clinical screening guidelines that PSA testing should not occur without shared decision-making between the clinician and patient^48^. The goal of prostate cancer screening is to identify those men with clinically significant localized disease who can be successfully treated, thereby preventing the morbidity and mortality associated with advanced or metastatic cancer. Identifying those men with low-risk, localized disease who can be managed appropriately using AS in order to avoid treatment-associated adverse effects is another key objective. Currently, 20–50% of men diagnosed with prostate cancer as a result of screening are likely to be overtreated^49^. These statistics highlight the importance of shared decision-making between men and their primary health-care professional, in which each individual man is independently assessed for his need to have PSA testing and the timing thereof, taking into account those men at an increased risk of developing prostate cancer owing to their family history and/or ethnicity (for example, men of Afro-Caribbean descent are at an increased risk of developing prostate cancer)^50^.

Growing evidence suggests that improved risk stratification using biomarker models can improve prostate cancer diagnosis. Numerous FDA-approved blood-based, urine-based and exosome-based diagnostic biomarkers (for example, Prostate Health Index^51^ and PCA3^52^) or CLIA-certified (such as 4 K score^53^, SelectMDX^54^ and ExoDx^55^) have demonstrated superiority to PSA testing in reducing the harms of prostate cancer testing. However, the population-wide adoption of these tests has faced a variety of challenges, including a need for extensive validation and cross-validation, different biomarker utilities in multiple clinical contexts and therapies, access and affordability issues, and a lack of head-to-head biomarker comparisons in prospective trials^56^.

Novel biomarkers with fewer false-positives and more sensitive detection of high-grade cancers than PSA are currently being tested in prospective, population-based trials. The STHLM3 study demonstrated a 32% reduction in the number of biopsies in 59,000 Scandinavian men who were tested with a panel of blood-based biomarkers compared with PSA testing alone, without loss in detection sensitivity^57^. Furthermore, in a meta-analysis comprising 46 clinical trials and 12,295 subjects evaluating urine *PCA3* mRNA levels, the marker was found to have good diagnostic performance (0.73 sensitivity, 0.65 specificity, 0.75 area under curve) in diagnosing prostate cancer^58^. The Prostate Health Index test is effective in cancer risk stratification in European and Asian men and different reference ranges have been developed for the different ethnic groups^59^. Presently, widespread clinical adoption and cost effectiveness in multiple jurisdictions are yet to be realized for these tests and future studies are, therefore, essential to ascertain whether these tests are clinically applicable across diverse populations and health-care systems.

Developments in imaging technology are demonstrating considerable improvements in the diagnosis, grading and monitoring of prostate cancer. High-quality evidence has shown that MRI can reduce the number of men who require a prostate biopsy and also reduce the diagnosis of clinically insignificant cancers that are unlikely to cause harm. A Cochrane systematic review and meta-analysis found that MRI with or without MRI-targeted biopsy compared with systematic transrectal ultrasonography-guided biopsy could increase true positive detection by 14% and decrease false-negative detection by 25% in men suspected of having prostate cancer^60^. In support of the use of imaging, the Prostate Imaging Reporting and Data System (PI-RADS) Steering Committee outlined how the MRI pathway should be incorporated into routine clinical practice, recommending that high-quality PI-RADS-compliant mpMRI should be performed before biopsy in most men suspected of having clinically important disease, who are likely to be offered active treatment^61^. mpMRI can provide multiple benefits over invasive transrectal ultrasound-guided biopsy, such as better distinguishing between clinically significant and insignificant tumours, a reduction in the number of biopsies performed and associated complications, improved biopsy accuracy, as well as potential cost savings^62,63^. mpMRI has been found to be both clinically effective and cost effective by the UK National Institute for Health and Care Excellence^64^. In the UK, 72% of men with suspected prostate cancer are currently being offered the scan before a biopsy^65^. Issues with interpretation and image quality, such as MRI-reading experience and lack of universally accepted technical quality criteria for prostate MRI are current barriers that still need to be addressed^61,66^. Future studies to understand the clinical effect that the implementation of MRI into routine workflows will have on the long-term health outcomes of men suspected of having prostate cancer are crucial.

The development, use and integration of imaging modalities with blood-based, tissue-based and urine-based biomarkers will also enable potential improvements in the identification and subsequent treatment of clinically significant lesions, as well as the detection and management of low-risk disease. The UK-based ReIMAGINE consortium^67^, which is aimed at integrating data from MRI and molecular biomarkers to further improve risk stratification and to reduce the overall costs of prostate cancer care, is currently investigating this possibility.

Establishing and adopting tests with improved sensitivity and specificity for prostate cancer screening and diagnosis might prove to be complex, but considerable benefit could be gained for men with prostate cancer and the wider health-care system, and this need should be a key area of future research focus.

### Research need number 2

#### Develop indicators to better stratify low-risk prostate cancer in determining which men should go on active surveillance

The concept of AS was first introduced in the early 2000s and has since become widely accepted as the standard of care for the management of men diagnosed with low-risk, localized prostate cancer^68^. In addition, it is postulated that AS might be a better option than aggressive clinical treatment for a subset of men with favourable, intermediate-risk disease, as it avoids the risk of detrimental urinary, bowel and sexual adverse effects of active therapies and consequently improves QOL^64^. Although several trials have been conducted in men with intermediate disease, further research incorporating the inclusion of advanced imaging modalities and biomarkers, which are increasingly being used to diagnose prostate cancer, are necessary to refine the criteria and better understand which men at intermediate risk should receive AS^69–71^.

Currently, men on AS are typically monitored using longitudinal evaluation of their serum PSA levels, imaging and tissue biopsies to ensure appropriate risk-classification (and re-classification) and, when necessary, selection for intervention if the disease has progressed^72^. However, a lack of consensus that defines this risk classification has led to substantial variation in AS protocols between and even within countries^73–75^. This variation is attributed to differences in risk stratification and inclusion criteria for AS, which are primarily based on a range of clinical assessments, which include Gleason grade pathology, imaging (mpMRI), and genomic assessments of tissue, blood and/or urine^76^. However, given that tissue biopsies commonly under-represent disease severity^77,78^, improvements in the accuracy of risk stratification and patient selection for AS are crucial. Molecular-based assays, such as Decipher, Oncotype, Prolaris and ProMark, are included in National Comprehensive Cancer Network guidelines and can be considered for men with low-risk (Gleason grade 3 + 3), or low-volume intermediate disease (Gleason grade 3 + 4) to improve risk stratification^79^ but have not yet been widely adopted. This lack of adoption is due to controversy regarding how to integrate data from these novel diagnostic tests into clinical practice and decision-making, as they were not originally developed for AS populations and might not be optimized for this setting. The use of mpMRI has become central in the management of prostate cancer and is included in many AS protocols to improve risk stratification and patient selection^80,81^. Studies have demonstrated that mpMRI-targeted biopsies are more accurate at detecting clinically significant disease with greater efficiency than biopsies alone and that detection of a lesion with mpMRI increases the likelihood of detecting high-risk disease at subsequent biopsy^82–84^. As such, mpMRI is used in several AS protocols to guide clinical decision-making. Selecting patients on AS using mpMRI remains a challenge with various screening measures and risk stratification methods currently in use, each with their own inclusion criteria and definitions of disease progression. Further studies on the optimization of mpMRI in the management and selection of men on AS are necessary.

In an effort to reduce the variation between AS protocols, a collective of prostate cancer-related European medical associations representing urology, nuclear medicine, radiotherapy, oncology and geriatric oncology formed a Prostate Cancer Guideline Panel and initiated a protocol-driven, three-phase study, in which consensus was achieved on 93 of 129 statements^85^. Consensus statements were formulated covering criteria as broad as patient selection, inclusion and exclusion criteria (including patient and disease characteristics, imaging and type of biopsy), the nature and timing of investigations and assessments during the period of monitoring and follow-up assessment (including PSA measurements, clinical examination, repeat imaging and repeat biopsies), criteria and thresholds for reclassification and change in management, and type of outcome measures to be prioritized^85^. Until higher levels of evidence emerge through prospective comparative studies, the findings from the Prostate Cancer Guideline Panel will be useful to inform routine clinical practice.

Despite AS being a viable option to safely reduce the overtreatment of low-risk disease, the continuous monitoring of men has been described as a considerable burden for many patients with repeat biopsy adherence decreasing over time, independent of biopsy frequency^86^. This observation highlights a fundamental need to develop more accurate, non-invasive and personalized approaches that are tailored to individual men on AS. Risk calculators, such as the Canary Prostate Active Surveillance Study risk calculation^87^, the Johns Hopkins model^88^ and the PRIAS model^89^ have been developed in an effort to address this need. These AS risk calculators can selectively predict men at risk of progression and balance reclassification detection with the number of surveillance biopsies by incorporating the serial measurement of the monitoring tools into the prediction model (for example, patient age, prostate volume, PSA, time since diagnosis, number of previous biopsies and all previous biopsy results)^90–94^.

Improvements in survivorship support, including a more dynamic and personalized approach to monitoring, could reduce cancer-related anxiety, which has been identified as a key factor in the discontinuation of AS^86^. The collection of patient-reported outcome measures from men with prostate cancer through local and global clinical registries will also be an important source of real-world evidence to improve the quality of care and optimal management of men undergoing AS. When large datasets are analysed and the knowledge disseminated accordingly, these patient-reported outcome measures will help to identify important considerations such as cancer-related anxiety that need to be addressed to ultimately improve QOL and clinical practice. It is anticipated that these registries will highlight potential areas of patient need where relevant strategies can be implemented to improve the care of patients.

The Movember-funded Global Action Plan 3 on AS (GAP3) was initiated to support ongoing research in this dynamic area. The retrospective data from over 20,000 men on AS have been included in the study to date, becoming one of the largest pooled prostate cancer AS cohorts in the world^95^. The GAP3 dataset has been used to report findings on protocol adherence for low-risk disease^96^, reasons for discontinuation of AS^97^, biopsy grading consistency^98^ and validation of AS reclassification calculators^99^.

### Research need number 15

#### Integration of companion diagnostics in randomized clinical trials to enable prediction of treatment response

Novel therapeutic options for advanced prostate cancer have dramatically increased during the past decade. Men with mCRPC have improved overall survival when treated with therapies that target the AR^100,101^. Emerging prostate cancer therapies such as immune checkpoint inhibitors and PARP inhibitors, which were recently approved by the FDA, also elicit clinical responses, but in smaller subsets of men with mCRPC than AR-targeting therapies^102,103^. These new agents for advanced prostate cancer could deliver other benefits to men (including response prediction, decreased adverse effects and decreased financial toxicity) if predictive biomarkers were used to guide therapy decisions. Men with advanced disease have increased volumes of material originating from their tumour in their circulation (such as circulating tumour cells (CTCs) and circulating tumour DNA (ctDNA))^104^, allowing blood-based biomarker assays to be developed. These liquid biopsies, including AR-V7 CTC test (Epic Sciences), CellSearch (Veridex) and Adna test (Qiagen) provide advantages over tissue biopsy as they are less invasive and can be routinely collected, enabling the real-time evaluation of tumour burden and genomic status^105^. This evaluation in real time is particularly relevant as, inevitably, tumours develop treatment resistance over time. Real-time monitoring enables identification of the development of tumour resistance, meaning that more informed second-line and third-line treatment decisions can be made.

An example of a predictive biomarker with demonstrated clinical utility is the commercially available AR-V7 test, which predicts the likelihood that a man with mCRPC will not benefit from the AR-targeted therapies enzalutamide and abiraterone, based on the expression level of the AR splice variant 7 mRNA in CTCs^106^. This test is being used by physicians in clinical practice and can be reimbursed through Medicare in the USA. The AR-V7 test has yet to receive FDA approval owing to a lack of consensus on the interpretation of the test and prospective evidence of overall survival^107^. AR-targeted therapies are now increasingly used in earlier stages of disease, including FDA approval for metastatic castration-sensitive and non-metastatic castration-resistant disease. To ensure that therapies remain effective, identifying and monitoring resistance with validated predictive biomarkers and incorporating these as companion diagnostics into prospective clinical trials to improve prediction of treatment response will be crucial.

The declining cost of sequencing technologies means that an unprecedented opportunity exists for personalized genomic profiling and tumour monitoring. Concordance between *AR* mutations in ctDNA and matched patient tissue biopsies is high, supporting the development of DNA biomarkers to guide the management of patients with mCRPC based on ctDNA alone^108^. A particular area of promise is the serial monitoring of AR alterations and *AR* mutation status that could enable the detection of treatment resistance before clinical progression^109^. Evaluation of ctDNA could help to define the role of homologous DDR mutations, which affect ~20% of patients with mCRPC and could be targeted using PARP inhibitors^110^. Germline mutations are readily detectable in leukocytes, but somatic mutations (reversion mutations and homozygous deletions) can be detected in ctDNA, enabling their clinical relevance to be delineated and the development of treatment resistance to be identified^111^. Hypermutations and microsatellite instability can also be detected in ctDNA and could be useful for predicting response to immune checkpoint inhibitor therapies; they are, therefore, promising biomarkers worthy of further exploration^112^.

Biomarker analysis is increasingly being implemented in the protocols of investigator-led trials (including STAMPEDE^113^, ProBIO^114^ and PC-BETS^115^) in order to improve treatment selection and monitor resistance. As liquid biopsy development continues, opportunities will become available to maximize multimodal approaches such as genetic sequencing and proteomic characterization of CTCs and exosomes, in order to increase the understanding of real-time tumour status^116^. CTC characterization and sequencing will be validated using ctDNA assessments in a longitudinal manner to improve understanding of tumour heterogeneity, enabling the optimal treatment of advanced prostate cancer. Enhanced collaboration between researchers, pharmaceutical companies, diagnostics companies, not-for-profit foundations and regulators, as well as increased patient engagement, will be essential for realizing these opportunities for the benefit of patients.

## Limitations

A number of limitations and caveats should be considered when interpreting this landscape analysis. First, the scope of this analysis was restricted to biomedical and clinical research areas and excluded survivorship research or clinical quality initiatives such as patient registries. Second, the prostate cancer treatment landscape is changing rapidly and the field has seen practice change in the past 3 years since the landscape analysis took place. Consequently, some research needs identified at the time of the analysis might not adequately reflect the current field. However, this report is current at the time of publication. Third, the identified research needs are probably most relevant in well-resourced health-care settings, where high-quality routine clinical care is standard, but could be less applicable to developing countries, as the landscape analysis was primarily designed to improve understanding of the research gaps and opportunities so that Movember can implement programmes in countries in which it currently fundraises and operates (which will hopefully also have a future positive impact on a global scale). Fourth, this landscape analysis was conducted by Movember as an organization that operates internationally; thus, country-specific nuances might not have been taken into consideration. Finally, some degree of overlap between certain closely linked research needs is inevitable. For example, a need for more accurate diagnostic tests might also have implications for research needs related to precision medicine, disease diagnosis and disease recurrence.

## Future directions

The LAC recommended that Movember focus its future prostate cancer biomedical research investments on research areas that lead to the early identification and optimal clinical management of men diagnosed with prostate cancer. This recommendation was proposed to the Movember GSC in February 2018.

The GSC agreed with the LAC’s recommendation and also felt that there was a need for Movember to continue to invest in translational research that will lead to an improved understanding of the biology of the disease and new or improved targets for CRPC, in order to enable optimal treatment of men who progress to advanced disease.

Moving towards its 2030 goal, Movember has combined the LAC’s and the GSC’s recommendations to establish two strategic priorities for future biomedical research investment. New investments will be made in highly translational research programmes that will lead to the earlier identification and optimal treatment of men with clinically significant disease in order to reduce the number of men progressing to advanced disease (strategic priority 1), as well as programmes with a focus on new or improved targets for CRPC that will lead to the optimal treatment of men who do progress to advanced disease (strategic priority 2). The rationale to invest in research related to strategic priority 1 is that an increased accuracy of risk stratification for clinically significant localized disease will support improved patient management, including optimized treatment decision-making. The rationale for further investment into research related to strategic priority 2 is that these efforts will eventually optimize the treatment of men who do progress to advanced prostate cancer.

These two strategic priorities have been endorsed by the Movember Board and Movember is now developing a range of collaborative research programmes across the globe to address these priorities, as well as many of the specific key research needs identified during the landscape analysis process. To ensure that its research investments reflect the needs of men and the research, clinical and societal community, Movember will continue to engage with its patient advocates and global thought leaders to maximize outcomes that positively change clinical practice and reduce economic burden for the benefit of the men that we serve.

## Conclusions

To identify the highest priority research needs across the prostate cancer biomedical research arena, Movember conducted a global landscape analysis. Using extensive interviews with men with prostate cancer as well as global thought leaders across a range of disciplines, key insights were ascertained and 17 critical research needs were identified. These research needs were assessed and prioritized by an expert LAC and the three highest ranked research needs are outlined in detail in this paper. The insights garnered from this landscape analysis have led Movember to refine its prostate cancer biomedical research strategic priorities and will inform future investments in the global research field with a view to maximizing outcomes for men with prostate cancer around the world.
